# Acceptability and feasibility of genital self-sampling for the diagnosis of female genital schistosomiasis: a cross-sectional study in Zambia

**DOI:** 10.12688/wellcomeopenres.15482.2

**Published:** 2020-09-02

**Authors:** Comfort Rutty Phiri, Amy S. Sturt, Emily L. Webb, Namakau Chola, Richard Hayes, Kwame Shanaube, Helen Ayles, Isaiah Hansingo, Amaya L. Bustinduy, BILHIV study team

**Affiliations:** 1 Zambart, Lusaka, Zambia; 2 Department of Clinical Research, London School of Hygiene & Tropical Medicine, London, WC1E 7HT, UK; 3 MRC Tropical Epidemiology Group, London School of Hygiene & Tropical Medicine, London, WC1E 7HT, UK; 4 Gynecology Department, Livingstone Central Hospital, Livingstone, Zambia

**Keywords:** female genital schistosomiasis, acceptability, feasibility, self-sampling, self-collection, vaginal self-sampling, cervical self-sampling, genital self-sampling

## Abstract

**Background**: Female genital schistosomiasis (FGS) is a neglected and disabling gynaecological disorder that is difficult to diagnose and is part of the wider spectrum of urogenital disease caused by the waterborne parasite *Schistosoma haematobium*. Over 90% of human schistosomiasis cases are found in sub-Saharan Africa with 3.8 million people infected with schistosomes in Zambia. Reported FGS prevalence ranges from 33-75% of those with urinary schistosomiasis in endemic areas, suggesting a potentially high FGS burden in Zambia alone. The Bilharzia and HIV

(BILHIV) study evaluated home self-sampling genital collection methods for the diagnosis of FGS.

**Methods**: Eligible participants included non-pregnant, sexually active women aged 18-31 who were previously recruited for the HPTN 071

(PopART) trial in Livingstone, Zambia. Household demographic and symptom questionnaires were administered by community workers. Participants were offered vaginal and cervical self-swabs and a urine cup. Cervicovaginal lavage (CVL) was performed in clinic by midwives. Information was collected from participants on the acceptability and feasibility of genital self-sampling.

**Results**: From January-August 2018, 603 women were enrolled, and 87.3% (527/603) completed clinic follow up. A high proportion of participants indicated that self-collection of specimens was “easy” or “very easy” on a 5-point Likert scale. A high proportion of women would be willing to self-collect all three specimens again in future: vaginal swab 96.7%

(583/603), cervical swab 96.5% (582/603), and urine 96.2% (580/603). Overall, 90.0% (543/603) preferred to self-collect samples at home, compared with sampling in the clinic Home-based self-sampling was preferred over provider-based sampling in the clinic due to greater privacy 65.0% (353/543), convenience 51.4% (279/543) and lack of needed transportation 17.7% (96/543).

**Conclusions**: Home based genital self-sampling for FGS diagnosis is highly acceptable. This scalable method may inform future efforts for community-based diagnosis of FGS

## Introduction

Human schistosomiasis is a waterborne parasitic disease caused by blood flukes of the genus *Schistosoma* ^1, 2^. It constitutes a significant public health problem causing the loss of 1,440 million years of full health worldwide, with approximately 659 million people at risk of acquiring infection ^2, 3^. More specifically, *Schistosoma haematobium* affects both the urinary as well as the genital tract. In female genital schistosomiasis (FGS) ^1^, parasite egg deposition occurs in the genital tract and it is characterized by histologic vaginal or cervical mucosal inflammation ^4^ and unique clinical findings ^5^. FGS has been associated with infertility, a condition associated with negative social and psychological impacts in many low-income countries ^6^. In addition, observational studies have suggested an association between FGS and prevalent HIV infection ^7, 8^, and HIV transmission and acquisition ^9^.

Genital self-sampling has been described in the diagnosis of reproductive tract infections (RTI) ^10– 12^ in both adults and adolescents ^13^ and has enhanced access to health services among hard-to-reach populations such as adolescents/young people ^14^, and those who do not regularly access health screening services ^15, 16^. A high proportion of women, including those from resource-limited settings have been found to prefer vaginal specimen self-collection ^10, 17^ compared with clinic-based sampling. In addition to acceptability, two other factors make genital self-sampling advantageous; 1) the availability of vaginal self-sampling is effective for improving participation in specific RTI screening programmes and 2) the sensitivity of PCR-based assays on self-collected specimens compares favourably with physician-performed sampling ^16, 18^.

The Bilharzia and HIV (BILHIV) study’s primary aim was to validate home-based self-sampling for the detection of *Schistosoma* DNA with vaginal and cervical swabs against provider obtained cervicovaginal lavage in a clinic setting in an endemic area in Zambia. The BILHIV study previously found that *Schistosoma* DNA was more frequently detected in genital self-collected specimens compared to clinic-collected cervicovaginal lavage ^19^. Here, we describe the acceptability and feasibility of genital self-sampling for the detection of *Schistosoma* DNA in the BILHIV study. In addition, this study also analyses the demographic predictors for participant’s preference of home-based self-sampling over clinic-based sampling.

## Methods

### Study setting and participants

The Bilharzia and HIV (BILHIV) study was a cross-sectional study nested within two of the 12 HPTN 071 (PopART) communities in Livingstone, southern province of Zambia ^20^. HPTN 071 (PopART) was a trial to measure the impact of an HIV combination prevention package, including universal test and treat ^20^. Non-pregnant, sexually active women aged 18-31 who had previously been recruited for the HPTN 071 (PopART) population cohort were eligible for inclusion in BILHIV.

### Sample collection and questionnaire

Between January and August 2018, specially trained population cohort research assistants visited women during the population cohort 36-month end of study follow up and enquired regarding an “expression of interest” in the BILHIV study. At a subsequent home visit, BILHIV Community Workers (BCW) evaluated study eligibility, provided participants with study information in the language of their choice, along with FGS education, and obtained written informed consent.

**Figure 1.**
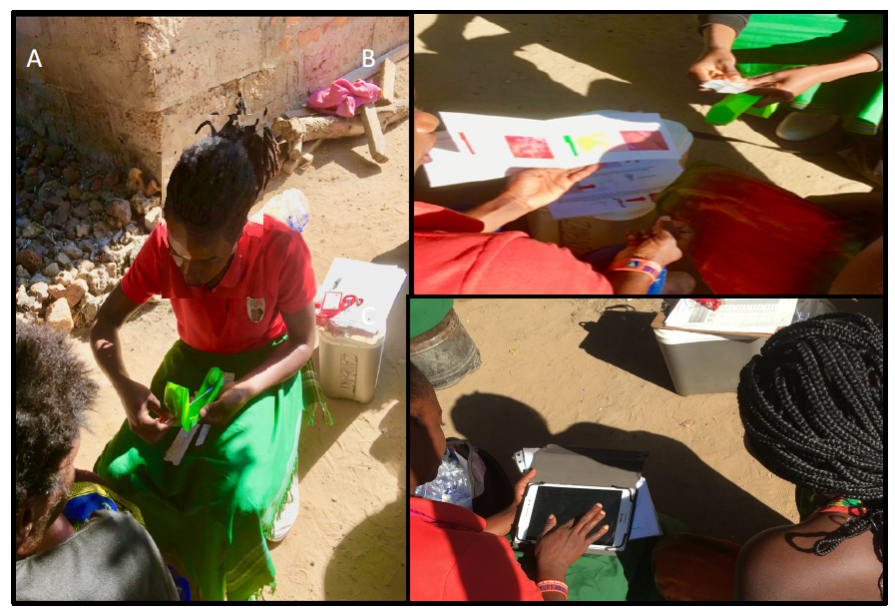
( **A**) The Bilharzia and HIV Community Workers (BCWs) demonstrating the use of genital self-swabs by using a 3D model; ( **B**) BCWs teaching by using the WHO female genital schistosomiasis atlas; ( **C**) BCWs delivering questionnaires in hand-held tablets. Photo credit: A. Bustinduy; oral permission was obtained from subjects to publish these images. Images have also been edited (pixelated and cropped) to keep the identity of the subjects anonymous.

At the home visit, conducted in each participant’s household, the BCW provided participating women with instructions for urine collection and cervical and vaginal self-swabs using educational materials including an information sheet with diagrams of the female anatomy, model vagina, and test swabs. Photos in the World Health Organization’s “Female Genital Schistosomiasis Pocket Atlas” were also displayed as a visual aid. As shown in Figure 1, these educational materials were used to explain and demonstrate the procedure of self-collection of genital specimens. For swab self-collection, participants were instructed to hold a 6-inch vaginal swab (PrimeSwab, Longhorn Diagnostics, Texas, USA) at the 2 3/8-inch score mark and insert the swab vaginally until their fingers touched the labia minora. Participants moved the swab in a circular motion against the vaginal walls for a minimum of 15 repetitions. Similarly, for the cervical swab, participants were instructed to hold a 6 3/4-inch flocked swab (Miraclean, Shenzen, China) with a quadrilateral kite-shaped tip at the non-flocked end of the swab body and insert the swab vaginally until they met noticeable resistance. The participant then performed swab rotation as described above. The participant broke the shaft of each swab and placed the vaginal and cervical swabs in separate screw-capped microtubes (STARLAB, Hamburg, Germany). Both swab specimens and urine were placed in cool boxes for transportation to the laboratory.

Following written informed consent and specimen collection, the participants completed a non-anonymous questionnaire, with responses captured on hand-held tablets. The questionnaire assessed basic demographics, information regarding genital symptoms, sexual behaviour and also the participant’s assessment of the acceptability of self-sampling, through their responses to 15 questions each measured on a five-point Likert scale ( *Extended data* ^21^; Table 1).

**Table 1.** Baseline characteristics of 603 Zambian women living in *Schistosoma haematobium* endemic areas near the Zambezi river by community.

Characteristics		Overall (n=603)	Community A (n=319)	Community B (n=284)	p-value *
Age in years – Median (IQR)		24 (22-28)	26 (23-29)	24 (21-27)	<0.001
Marital Status	Single	258 (42.8%)	110 (34.5%)	148 (52.1%)	<0.001
	Married or Cohabitating	320 (53.1%)	193 (60.5%)	127 (44.7%)	
	Divorced or Separated	23 (3.8%)	15 (4.7%)	8 (2.8%)	
	Widowed	2 (0.3%)	1 (0.3%)	1 (0.4%)	
Education (highest level)	Any Primary School	167 (27.7%)	117 (36.7%)	50 (17.6%)	<0.001
	Any Secondary School	364 (60.4%)	173 (54.2%)	191 (67.3%)	
	Training in a Trade	59 (9.8%)	20 (6.3%)	39 (13.7%)	
	Degree or Higher	3 (0.5%)	3 (0.9%)	0 (0.0%)	
	None	10 (1.7%)	6 (1.9%)	4 (1.4%)	
Employment status	Working	408 (67.7%)	200 (62.7%)	208 (73.2%)	0.006
	Not Working	195 (32.3%)	119 (37.3%)	76 (26.8%)	
Current water contact	None	512 (84.9%)	263 (82.5%)	249 (87.7%)	0.02
	At Least Weekly	18 (3.0%)	11 (3.5%)	7 (2.5%)	
	Every 1–2 Months	30 (5.0%)	24 (7.5%)	6 (2.1%)	
	Every 6–12 Months	43 (7.1%)	21 (6.6%)	22 (7.8%)	
Childhood water contact	None	186 (30.9%)	96 (30.1%)	90 (31.7%)	0.22
	At Least Weekly	381 (63.2%)	208 (65.2%)	173 (60.9%)	
	Every 1–2 Months	24 (4.0%)	12 (3.8%)	12 (4.2%)	
	Every 6–12 Months	12 (2.0%)	3 (0.9%)	9 (3.2%)	
	No	572 (94.8%)	294 (92.2%)	278 (97.9%)	0.006
Self-reported history of schistosomiasis	Yes	25 (4.2%)	20 (6.3%)	5 (1.8%)	
	Maybe	6 (1.0%)	5 (1.6%)	1 (0.4%)	

*comparison of Community-A vs Community-B

At a later date, participating women who were not currently menstruating attended Livingstone Central Hospital (LCH) cervical cancer screening clinic where a trained midwife performed a cervicovaginal lavage and images of the vagina and cervix were captured with a point-of-care colposcope (MobileODT, Tel Aviv Israel) ^19^

### Ethics and informed consent

All eligible participants providing written consent were recruited into the study. Participants who were unable to provide written informed consent were recruited in the presence of a witness with the participant placing their thumbprint on the consent form. The study was approved by the University of Zambia Biomedical Research Ethics Committee (reference number: 011-08-17), the Zambia National Health Research Authority and the London School of Hygiene and Tropical Medicine research ethics committee (reference number: 14506). Permission to conduct the study was given by the Livingstone District health office and the superintendent of the Livingstone Central Hospital.

### Data management and statistical methods

Acceptability in our study was measured by the following outcomes: the proportion of women who rated home based self-sampling to be “ *easy” or “very easy”* (for each of urine, vaginal, cervical self-sampling), the proportion who didn’t experience “ *pain”* while self-sampling (for each of vaginal, cervical self-sampling), the proportion who were willing to self-sample again *“in the future”* (for each of urine, vaginal, cervical self-sampling), and the proportion who would prefer to “ *sample at home”* (versus sampling in the clinic).

Participant data were entered using Open Data Kit Collect ^22^. Continuous variables were summarized by mean and interquartile range (IQR), and categorical variables by frequency and percentage. Participant characteristics were compared between the two communities using Wilcoxon-Mann-Whitney, chi-squared, and Fisher’s exact tests. The Mantel-Haenszel approach was used to obtain crude and age-adjusted odds ratios for the association of demographic variables with a participant’s preference for home-based versus clinic-based sampling.

## Results

**Figure 2.**
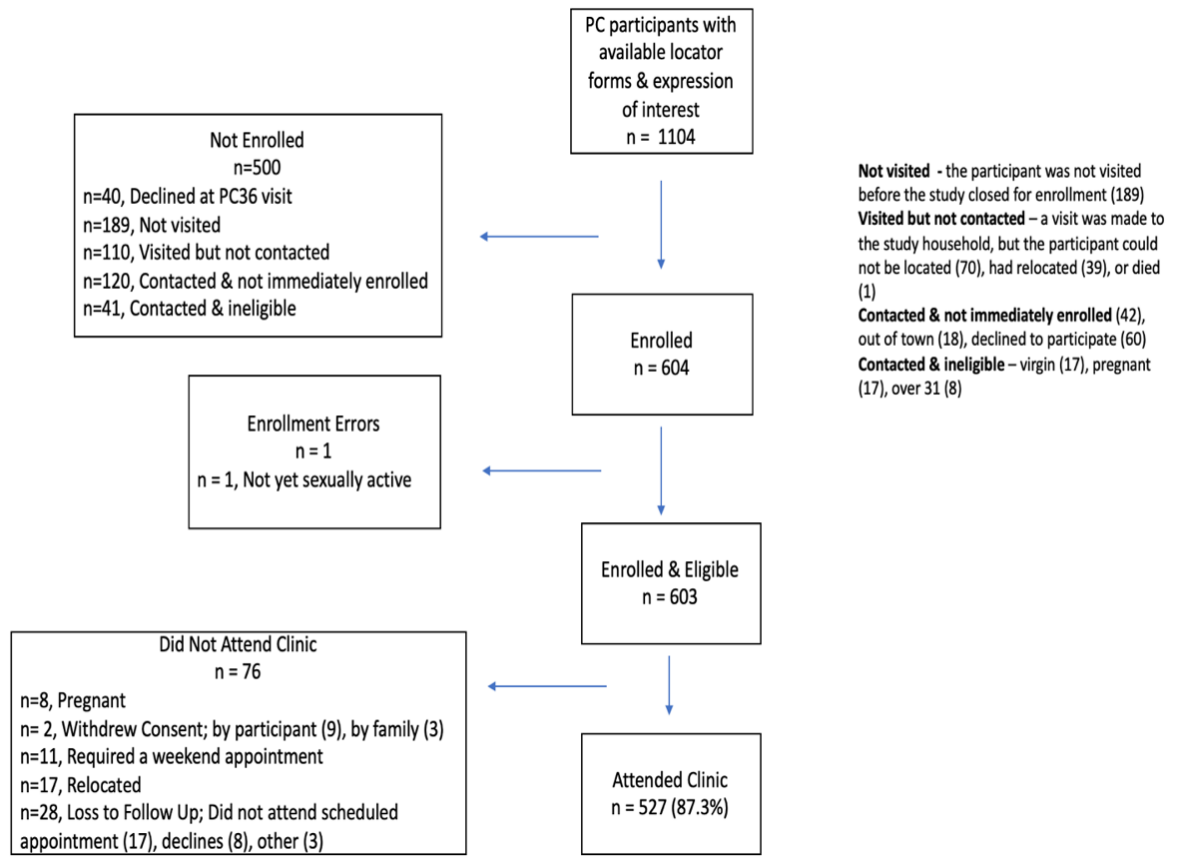
The Bilharzia and HIV study enrolment and sampling flow chart.

Of 1104 women screened for BILHIV eligibility, 54.5% (603/1105) were enrolled and all completed an initial home-based visit. Of those completing the initial home visit, 87.4% (527/603) completed clinic follow up visit ( Figure 2). Unless otherwise stated, the denominator for the results presented reflects the total study enrolment of 603. The median age was 24 years (IQR 22-28). More than half of participants, 60.4% (364/603), completed secondary school education and 59% (356/603) spoke primarily Nyanja ( Table 1). Active schistosome infection was determined by detectable urine Circulating Anodic Antigen (CAA) (15.1%, 91/601) or microscopy (5.5%, 33/603), as previously described ^18^. Compared to clinic-collected CVL (14/527, 2.7%), *Schistosoma* DNA was more frequently detected in genital self-collected specimens (24/603, 4.0%) ^19^.

### Acceptability and feasibility

**Figure 3.**
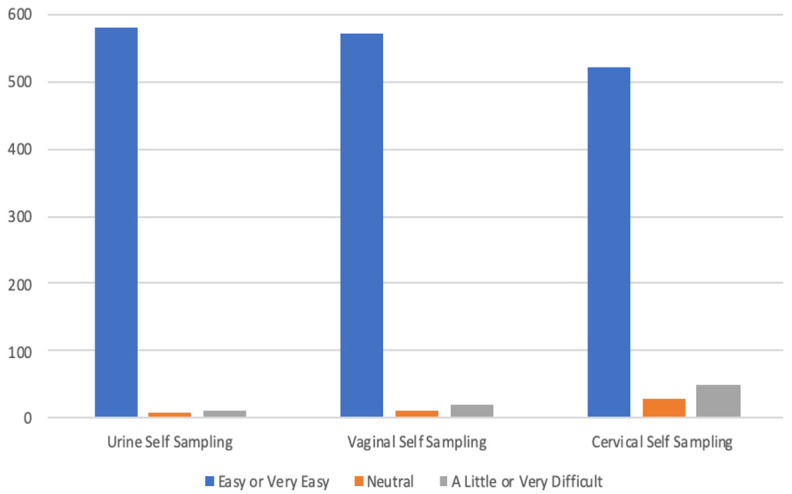
Ease of self-sampling in 603 Zambian women by specimen type.

Out of 603 women recruited, a high proportion indicated that self-collection of genital specimens was “ *easy*” or “ *very easy*” on a 5-point Likert scale for urine collection (96.2%; 580/603), vaginal swab (94.9%; 572/603), and cervical swab (86.6%; 522/603) ( Figure 3; Table 2). Most participants indicated that they would be willing to self-collect again in the future: urine 97.0% (585/603), vaginal swab 96.7% (583/603) and cervical swab 96.5% (582/603). Substantially less than half of participants reported that it was “ *painful”* to self-collect vaginal specimens (3.3%; 20/603) and cervical specimens (6.8%; 41/603) ( Table 2). A high proportion of women (95.7%; 577/603) indicated that they would ‘ *recommend self-sampling to my friends*’. Overall, most women preferred to collect specimens at home (90.0%; 543/603), compared with clinic-based sampling (10.0%; 60/603), ( Table 3). Women from both communities preferred to self-collect specimens from home (Community A: 89.3%, 285/319; Community B: 90.9%, 258/284; p=0.5) compared with attending the health facility. Participants preferred “ *self-sampling at home”* over provider-based sampling in the clinic due to greater privacy (65.0%, 353/543), convenience (51.4%, 279/543) and lack of transportation (17.7%, 96/543) ( Table 3). Participants in Community B were more confident (99.3%; 282/284) than participants in Community A (91.5%; 292/319) (p<0.001) that they collected the specimens correctly.

**Table 2.** Acceptability of genital self-sampling for women from the BILHIV study (n=603).

Question	Very easy % (n)	Easy % (n)	Neutral % (n)	A little difficult % (n)	Very difficult % (n)
I found vaginal self-sampling to be	34.5 (208)	60.4 (364)	2.0 (12)	3.2 (19)	0 (0)
I found cervical self-sampling to be	26.2 (158)	60.4 (364)	5.0 (30)	8.5 (51)	0 (0)
I found collecting my own urine sample to be	56.2 (339)	40.0 (241)	1.7 (10)	2.0 (12)	0.2 (1)
Question	Strong yes	Yes	Maybe	No	Strong no
I would be willing to take a vaginal self-sample in the future.	42.1 (254)	54.6 (329)	2.2 (13)	1.2 (7)	0 (0)
I would be willing to take a cervical self-sample in the future.	37.0 (223)	60.0 (359)	2.5 (15)	1.0 (6)	0 (0)
I would be willing to takes a urine self-sample in the future.	38.6 (233)	58.4 (352)	2.3 (14)	0.7 (4)	0 (0)
I would recommend self-sampling to my friends.	29.0 (175)	66.7 (402)	1.8 (11)	2.0 (12)	0.5 (3)
Self-collecting a vaginal swab was painful.	0.33 (2)	3.0 (18)	3.7 (22)	77.1 (465)	15.9 (96)
Self-collecting a cervical swab was painful.	0 (0)	6.8 (41)	9.6 (58)	71.3 (430)	12.3 (74)
I am confident I collected the specimens properly.	29.0 (175)	66.2 (399)	2.7 (16)	2.2 (13)	0 (0)
I feel confident I collected a sample from my vagina.	25.7 (155)	72.3 (436)	1.3 (8)	0.7 (4)	0 (0)
I feel confident I collected a sample from my cervix.	24.5 (148)	71.6 (432)	3.5 (21)	0.3 (2)	0 (0)

**Table 3.** Results of the BILHIV study patient experience surveys for 603 women living in *Schistosoma haematobium* endemic areas in Livingstone, Zambia*.

Question	Participant responses	% (n) *
Do you prefer to take your samples at home, or would you prefer to take samples at the clinic?	Clinic	10.0 (60)
	Home	90.0 (543)
I prefer doing samples at home because **	It is more convenient	51.4 (279)
	I don’t have transportation	17.7 (96)
	I don’t have childcare	2.6 (14)
	I need to work	6.2 (34)
	I have more privacy at home	65.0 (353)
	It is easier to sample at home	66.3 (360)
	Other reason	11.4 (62)
I prefer having samples performed in clinic because **	I don’t have privacy at home	26.7 (16)
	I had discomfort with collecting my own samples	13.3 (8)
	I was unsure if I did the sampling properly	30.0 (18)
	I’d like more supervision	28.3 (17)
	Other	28.3 (17)

*Proportions for home-based testing have a denominator of 543, proportions for clinic-based testing have a denominator of 60 **Participants could choose more than one answer

Overall, there was little evidence that education, marital status, community of residence, employment status, language spoken, and age were associated with a participant’s preference for home-based sampling over clinic-based sampling ( Table 4). Given that the preference for self-sampling was universal across the groups examined in the crude analysis, we did not undertake multivariable analysis.

**Table 4.** Factors associated with the choice of home-based sampling over clinic-based sampling, adjusted for age.

Exposure		n (home-based sampling)/N (%)	Crude OR	95% CI	aOR	95% CI	p-value
Education	None or any primary school	166/177 (94%)	reference		reference		0.31
	Any secondary school	323/364 (89%)	0.52	0.26 – 1.05	0.45	0.22 – 0.91	
	Trade training or a degree	54/62 (87%)	0.45	0.17 – 1.18	0.47	0.17 – 1.27	
Language*	Nyanja	328/356 (92%)	reference		reference		0.11
	Tonga	114/127 (90%)	0.75	0.37 – 1.50	0.75	0.38 – 1.52	
	Lozi	72/86 (84%)	0.44	0.22 – 0.88	0.44	0.22 – 0.88	
	Bemba	26/30 (87%)	0.55	0.18 – 1.71	0.55	0.18 – 1.70	
Marital status	Single	228/258 (88%)	reference		reference		0.49
	Married	292/320 (91%)	1.37	0.79 – 2.37	1.58	0.85 – 2.95	
	Divorced or widowed	23/25 (92%)	1.51	0.34 – 6.77	1.61	0.31 – 8.34	
District	Community A	285/319 (89%)	reference		reference		0.54
	Community B	258/284 (91%)	1.18	0.69 – 2.03	1.14	0.66 – 1.97	
Employment status	Not working	367/408 (90%)	reference		reference		0.91
	Working	176/195 (90%)	1.03	0.58 – 1.84	1.07	0.60 – 0.90	
Age (years)	18–22	144/158 (91%)	reference		--	--	0.62
	23–26	207/228 (91%)	0.96	0.47 – 1.95	--	--	
	27–31	192/217 (89%)	0.75	0.37 – 1.49	--	--	

## Discussion

Vulnerable women and girls in sub-Saharan Africa are afflicted by FGS, a chronic gynaecologic condition. Current diagnostic strategies are limited as they rely on resources that are seldom available in low-income settings ^23^. A self-collection method that minimises reliance on health care providers would represent a scalable alternative method for FGS community-based diagnosis in endemic resource limited settings, but only if it is an acceptable procedure to perform. However, barriers to FGS diagnosis still remain, including costs, limited access to point-of-care diagnostics, and challenges with maintaining the cold chain. The cost of genital swabs (0.50$/vaginal swab and 0.30$/cervical swab) and molecular testing (6.68$/test) may be affordable in some research settings, but more field-appropriate and scalable methods should be investigated. Home based genital self-sampling for the diagnosis of FGS was highly acceptable among women aged 18 to 31 years of age enrolled in the BILHIV study in Zambia. All participating women provided all three self-collected specimens (urine, vaginal and cervical swabs), and a high proportion found vaginal self-sampling and cervical self-sampling “ *easy*” or “ *very easy*”.

Our study is in agreement with other studies in which self-swabs were acceptable to women in geographically and ethnically diverse target populations ^10, 18, 24^. In a study of Haitian immigrants living in the USA, the acceptability of unsupervised cervical HPV self-sampling using written instructions revealed that self-sampling was more acceptable to the majority of the women than clinician-administered sampling ^24, 25^, and it increased screening coverage among female clinic non-attendees ^15, 26^. Also in an Italian study, cervical self-sampling using either a brush or a self-lavaging device was acceptable and both modalities were preferred to clinician-sampling (n=117, 68%) ^27^. A systematic review on the acceptability of self-sampled screening for HPV DNA reported that self-sampling was highly acceptable among study participants in 37 studies from 24 countries across five continents ^25^. Despite heterogeneity in study design, the studies in this meta-analysis suggest that self-sampling is well accepted by participants regardless of education, marital status, community of residence, employment status, language spoken, and age. Supported by these data we can conclude that our findings are likely generalizable across geographic areas and among women of varying educational background, cultures, and ethnic groups.

Substantially over half of the women in the BILHIV study reported that self-collection of specimens was “ *easy” or “very easy”* (urine 96.2%, vaginal swab 94.9% and cervical swab 86.6%). This is consistent with other studies that showed that study participants found genital self-sampling or the use of a self-sampling device easy to use ^24, 25^. The proportion with this outcome was slightly lower for cervical than vaginal sampling. Swab length and more invasive technique may account for the lower proportion of women who found cervical self-sampling “ *easy*” or “ *very easy*”, compared with vaginal self-sampling. As another measure of acceptability, over 96% of women in the BILHIV study indicated that they were willing to self-collect *all three specimens* again in the future, which is similar to proportions reported in HPV self-collection research using cervical swabs ^24, 28^ and curable STI research using vaginal swabs ^29^. Our study, as others, further showed that a high proportion of the women indicated that they would recommend self-sampling to a friend ^25^. This shows promise for the future use of peer-encouragement in the use of genital self-sampling procedures.

Our study also revealed that 90.0% of participants preferred self-sampling at home over provider-based sampling at the clinic. Our findings are similar to studies reporting a high preference for home self-sampling ^25, 27, 28^. However, a recent meta-analysis found that the pooled estimate of women who preferred self-sampling to clinic based sampling was 59% (48 – 69%) ^25^. There are some possible explanations for this. While a binary outcome was evaluated in the meta-analysis, the individual reasons for preferring home-based self-sampling to health-facility sampling vary across studies. In the BILHIV study questionnaire, the questions regarding preferences for home vs. clinic sampling included a comprehensive range of options that included ‘privacy’, ‘convenience’, ‘transportation’, ‘work conflicts’, ‘no child-care’, and ‘ease’ among others. Second, other work reports that some women preferred clinic sampling to home based self-sampling because they were not comfortable with touching their genital areas, they were unsure about the safety of self-testing, or they were concerned they would perform the test incorrectly ^30^.

This study benefited from HPTN 071 (PopART) because HPTN 071 (PopART) staff introduced the BILHIV study to all prospective BILHIV participants that enabled them to be familiar with the study even before it began. Further, the BILHIV study was implemented in communities that were already familiar with the organization and the staff that worked under the HPTN 071 (PopART) study. In addition, former HPTN 071 (PopART) staff in the two study communities continued to work in the same communities under the BILHIV study. This enabled improved study performance because of the existing rapport between BILHIV staff and the community members. Standardized questionnaires were used to reduce observer bias and were performed at the time of self-sampling to minimize recall bias. However, it is important to note that the participation in the BILHIV study was limited to women who took part in the HPTN 071 (PopART) population cohort. In this scenario, bias may be related to a Hawthorne effect. This observer effect can occur as participants in a study alter their behaviour as a result of regular follow-up within a cohort ^31^. The HPTN 071 (PopART) population cohort was selected through a random sampling of households and random selection of one individual within each household ^31^. BILHIV study participants were selected by querying eligible members of the population cohort for an “expression of interest”. There may be selection bias, in that women who expressed an interest in participating in the study may not be representative of the population as a whole and findings may not be generalizable to other sub-Saharan African communities. A larger study of genital self-sampling should be performed, preferably in areas of varied schistosomiasis endemicity.

## Conclusion

We have shown high acceptability and feasibility of genital self-sampling for the diagnosis of FGS in young women (18–31 years) in a schistosomiases endemic area in Zambia. This practice has potential to increase FGS surveillance in other endemic populations. The majority of participants reported that specimen self-collection was “ *easy*” or “ *very easy*” with high willingness to participate in future home-based self-sampling. Results can inform future efforts for community-based diagnosis of FGS.

## Data availability

### Underlying data

LSHTM Data Compass: BILHIV acceptability dataset, https://doi.org/10.17037/DATA.00001618 ^32^.

This data is under restricted access due to the assurance given to participants that responses would be kept completely confidential. This is particularly important due to the sensitivity of the data produced. The data set can be accessed by completing the Request Form, which requires that the intended use for the data is specified. Data available under the LSHTM Data Compass Data Sharing Agreement.

### Extended data

Figshare: Extended data_Figshare.docx, https://doi.org/10.6084/m9.figshare.12023382.v1 ^21^.
