# Supplementary material for: Acceptability and feasibility of genital self-sampling for the diagnosis of female genital schistosomiasis: a cross-sectional study in Zambia
Source: Wellcome Open Res. 2020 Sep 2;5:61. Originally published 2020 Apr 2. [Version 2] doi: 10.12688/wellcomeopenres.15482.2 (PMC7610177; doi:10.12688/wellcomeopenres.15482.2)
Supplement: Peer review history

# Peer review: Reviewer response for version 2

**DOI:** 10.21956/wellcomeopenres.17751.r40248

**Published:** 2020-10-15

**Authors:** Peter Leutscher

**Affiliations:** 1 Center for Clinical Research, North Denmark Regional Hospital, Hjørring, Denmark

The manuscript is now approved from my part. Congratulations to the team.

Is the work clearly and accurately presented and does it cite the current literature?

Yes

If applicable, is the statistical analysis and its interpretation appropriate?

Yes

Are all the source data underlying the results available to ensure full reproducibility?

Yes

Is the study design appropriate and is the work technically sound?

Yes

Are the conclusions drawn adequately supported by the results?

Yes

Are sufficient details of methods and analysis provided to allow replication by others?

Yes

Reviewer Expertise:

NA

I confirm that I have read this submission and believe that I have an appropriate level of expertise to confirm that it is of an acceptable scientific standard.

# Peer review: Reviewer response for version 2

**DOI:** 10.21956/wellcomeopenres.17751.r40247

**Published:** 2020-09-09

**Authors:** Alexander Odaibo

**Affiliations:** 1 Parasitology Research Unit, Department of Zoology, University of Ibadan, Ibadan, Nigeria

The authors have clarified and addressed the reservations I had in my first review.

Is the work clearly and accurately presented and does it cite the current literature?

Yes

If applicable, is the statistical analysis and its interpretation appropriate?

I cannot comment. A qualified statistician is required.

Are all the source data underlying the results available to ensure full reproducibility?

Yes

Is the study design appropriate and is the work technically sound?

Yes

Are the conclusions drawn adequately supported by the results?

Yes

Are sufficient details of methods and analysis provided to allow replication by others?

Partly

Reviewer Expertise:

Epidemiology and control of schistosomiasis.

I confirm that I have read this submission and believe that I have an appropriate level of expertise to confirm that it is of an acceptable scientific standard.

# Peer review: Reviewer response for version 1

**DOI:** 10.21956/wellcomeopenres.16935.r39268

**Published:** 2020-07-20

**Authors:** Alexander Odaibo

**Affiliations:** 1 Parasitology Research Unit, Department of Zoology, University of Ibadan, Ibadan, Nigeria

The authors intended to evaluate the acceptability and feasibility of genital self-sampling for female genital schistosomiasis in a given cohort in Zambia. The intention is good and the execution is appropriate but there are a few clarifications to be made.

 Methods: 

1. No information was provided on the number of participants recruited for the study.
2. It is not clear from the article if there was a common sample collection centre for all participants or sample collection was done at the residence of each participant.
3. What was the *Schistosoma haematobium* infection status of the participants at the time of study?

Results:

1. Figure 2 is superfluous and what is the relevance of the table under result?
2. No information on how the home-based sampling by the women compared with the clinic sampling done in this study, instead readers are referred a paper that is still under review.

Discussion:

1. The authors may need to rephrase the first sentence under discussion to avoid starting the sentence with an abbreviation (FGS).
2. **Schistosomiases** should be changed to **schistisomiasis**.

Is the work clearly and accurately presented and does it cite the current literature?

Yes

If applicable, is the statistical analysis and its interpretation appropriate?

I cannot comment. A qualified statistician is required.

Are all the source data underlying the results available to ensure full reproducibility?

Yes

Is the study design appropriate and is the work technically sound?

Yes

Are the conclusions drawn adequately supported by the results?

Yes

Are sufficient details of methods and analysis provided to allow replication by others?

Partly

Reviewer Expertise:

Epidemiology and control of schistosomiasis.

I confirm that I have read this submission and believe that I have an appropriate level of expertise to confirm that it is of an acceptable scientific standard, however I have significant reservations, as outlined above.

# Response

**Published:** 2020-08-24

**Authors:** Amaya Bustinduy

**Affiliations:** London School of Hygiene & Tropical Medicine, UK

We thank Prof Odalbo for his helpful comments. To respond to his queries, this is a point by point response.

- No information was provided on the number of participants recruited for the study.

We apologize for any confusion, the information about total number of participants recruited can be found in the “Abstract” and also on page 6 under “Results” in the 1 ^st^ and 3 ^rd^ sentences in our final submitted manuscript. For convenience, we have highlighted this in the accompanying manuscript.

- It is not clear from the article if there was a common sample collection centre for all participants or sample collection was done at the residence of each participant.

Thank you for this input. We have added your point regarding sample collection was done on method section, sub section “Sample Collection and Questionnaire” line 6.

- What was the Schistosoma haematobium infection status of the participants at the time of study?

In the “Results” section we have now included the schistosome infection status of the participants in the study. We used both urine microscopy and Circulating Anodic Antigen (CAA). As the CAA is not species-specific, we have expressed infection status as “active schistosome infection”.

**Results:**

- Figure 2 is superfluous and what is the relevance of the table under result?

Thank you for your review. Figure 2 is the BILHIV Study Flow Diagram. We thought providing this information to readers might provide transparency regarding the included participants and enhance interpretation of the study’s generalisability.

 In terms of the tables in the “Results” section:

 Table 1 describes the baseline characteristics of 603 study participants

 Table 2 give more information regarding experiences women had during self-sampling.

 Table 3 describes the results of the patient experience surveys for 603 study participants

 Table 4 describes the demographic factors associated with the choice of home-based sampling over clinic-based sampling We feel these tables should be maintained.

- No information on how the home-based sampling by the women compared with the clinic sampling done in this study, instead readers are referred a paper that is still under review.

Thank you very much for bring up this point and for any inconvenience. In the third paragraph of the “Introduction” we describe that “the BILHIV study found that *Schistosoma* DNA was more frequently detected in genital self-collected specimens compared to clinic-collected cervicovaginal lavage”. At the time this manuscript was originally submitted we had not yet published the main results for the BILHIV study, however the paper that was under review is now published and it is cited as reference number 19.

**Discussion:**

- The authors may need to rephrase the first sentence under discussion to avoid starting the sentence with an abbreviation (FGS).

Thank you for this input. We have rephrased the sentence starting with abbreviation FGS under “Discussion”, line 1.

- Schistosomiases should be changed to schistisomiasis.

​​​​​​​Thank you for your observation. We have changed ‘Schistosomiases to schistosomiasis’ under “Conclusion” in line 2.

# Peer review: Reviewer response for version 1

**DOI:** 10.21956/wellcomeopenres.16935.r38334

**Published:** 2020-07-03

**Authors:** Peter Leutscher

**Affiliations:** 1 Center for Clinical Research, North Denmark Regional Hospital, Hjørring, Denmark

In this manuscript, results from questionnaire in a urogenital self-sampling study are presented addressing schistosomiasis infection in women aged 18-31 living in Zambia.

 Overall, the conclusion is clear. Self-sampling is very well accepted by the women, and apparently with only minor discomfort/distress, if any. This approach of home sampling as an alternative to urogenital sampling in a clinical setting seems attractive by providing the women elements of individual convenience and privacy.

 However, as also presented in the discussion, the study findings may not necessarily translate to the same extent into other communities in sub-Saharan Africa, mainly because a bias being potentially implicated due to previous study activities taking place in the populations before the self-sampling study. Therefore, another study should be performed, and preferable in different schistosomiasis endemic communities in Southern Africa to control for the potential bias.

 Other comments:

- Inclusion criteria (e.g age, non-pregnancy) are not stated in Methods.
- Why inform about a pending paper by Stuart *et al.*? More interesting to know about a probably pending paper presenting the lab findings (SH DNA).
- A 5-point Likert scale has been used. This information should be presented in the Methods and not in the Results.
- No information whether the questionnaire was performed anonymously or not, apparently not if one looks at the field image. Then there would have been a unique opportunity to uncover various reasons for reported lack of confidence, acceptance, comfort etc – even only reported in a minority of the women. 
- Is information about the different dialects in Table 1 of interest for the reader?
- Interesting that 32.3% of women are given the status as “Not working”.
- Information about Childhood water contact seems not that relevant (recall-bias) in adult women.
- How has self-reported history of schistosomiasis been assessed?

**Results **

- N=603 stated 17 times. Should be adequate to mention once, the number of study participants.
- Confusing that the percentages in Table 3 (I don’t have transportation, I have more privacy at home) are different from those in the main text (17.7% vs 15.9%; 65.0% vs. 58.5).

**Discussion **

- *FGS is a chronic gynaegological condition that afflicts vulnerable women*… What is meant by “vulnerable”?
- *Current diagnostic strategies are limited as they rely on resources that are seldom available in low-income settings. S*elf-sampling does not contribute significantly to solving this problem, only to minor extent, having the women to perform the sampling themselves instead of a health care provider (e.g a nurse). Other major cost will remain, including lab technicians, equipment, reagents etc.
- Participants should always be acknowledged.

Is the work clearly and accurately presented and does it cite the current literature?

Yes

If applicable, is the statistical analysis and its interpretation appropriate?

Yes

Are all the source data underlying the results available to ensure full reproducibility?

Yes

Is the study design appropriate and is the work technically sound?

Yes

Are the conclusions drawn adequately supported by the results?

Yes

Are sufficient details of methods and analysis provided to allow replication by others?

Yes

Reviewer Expertise:

NA

I confirm that I have read this submission and believe that I have an appropriate level of expertise to confirm that it is of an acceptable scientific standard, however I have significant reservations, as outlined above.

# Response

**Published:** 2020-08-20

**Authors:** Amaya Bustinduy

**Affiliations:** London School of Hygiene & Tropical Medicine, UK

In this manuscript, results from questionnaire in a urogenital self-sampling study are presented addressing schistosomiasis infection in women aged 18-31 living in Zambia.

Overall, the conclusion is clear. Self-sampling is very well accepted by the women, and apparently with only minor discomfort/distress, if any. This approach of home sampling as an alternative to urogenital sampling in a clinical setting seems attractive by providing the women elements of individual convenience and privacy.

However, as also presented in the discussion, the study findings may not necessarily translate to the same extent into other communities in sub-Saharan Africa, mainly because a bias being potentially implicated due to previous study activities taking place in the populations before the self-sampling study. Therefore, another study should be performed, and preferable in different schistosomiasis endemic communities in Southern Africa to control for the potential bias.

**Thank you for this input. We have added your point regarding performing other studies in schistosomiasis endemic communities in different regions to the discussion. Lines 67-70 under discussion.**

Inclusion criteria (e.g age, non-pregnancy) are not stated in Methods.

**We apologize for any confusion. In the BILHIV study, women were eligible if they were sexually active aged 18-31 who were not pregnant and had previously been recruited for the HPTN 071 (PopART) population cohort were eligible for inclusion in BILHIV. In the on-line version ( https://wellcomeopenresearch.org/articles/5-61) the inclusion criteria are stated in the first sentence of the abstract methods. In the main manuscript, the inclusion criteria are stated in the Methods section in the second sentence methods under “study setting and participants”. In the tracked-changes manuscript we have highlighted these areas for clarity.**

Why inform about a pending paper by Stuart et al.? More interesting to know about a probably pending paper presenting the lab findings (SH DNA).

**We agree that the section in question should be modified. In the section “Sample collection and questionnaire” in the final sentence, we have removed the wording “Sturt, A *et al.* paper under review” and instead we reference the BILHIV study manuscript. As you have suggested, this manuscript provides the full laboratory results.**

A 5-point Likert scale has been used. This information should be presented in the Methods and not in the Results.

**We apologize for any confusion, the information about the Likert scale was included in the methods section in our final submitted manuscript, but it seems not to have been uploaded into the online manuscript. We have highlighted this in the accompanying manuscript.**

No information whether the questionnaire was performed anonymously or not, apparently not if one looks at the field image. Then there would have been a unique opportunity to uncover various reasons for reported lack of confidence, acceptance, comfort etc – even only reported in a minority of the women. 

**You are correct that the questionnaire was not performed anonymously. We have clarified this in the methods.**

Is information about the different dialects in Table 1 of interest for the reader?

**Thank you for this input, although we agree with the reviewer that dialects are not directly of importance to the study outcome, we would like to keep them in the table as they contribute to a more holistic appreciation of the study participants and their background. This may be of interest for certain readers.**

Interesting that 32.3% of women are given the status as “Not working”.

**We agree that this is interesting. The women were asked the question “are you currently working?” with yes/no answer. These data reflect their self-reported response to this question.**

Information about Childhood water contact seems not that relevant (recall-bias) in adult women.

**We agree that the variable regarding childhood water contact is subject to recall bias. However, we thought this information would provide the reader with information regarding the participant’s perceived level of exposure.**

How has self-reported history of schistosomiasis been assessed?

**The self-reported history of schistosomiasis was not further assessed beyond the participant’s self-report.**

**RESULTS**

N=603 stated 17 times. Should be adequate to mention once, the number of study participants.

**Thank you for this input. We have repeated the total enrollment number to clarify the denominator for many of the presented proportions.**

Confusing that the percentages in Table 3 (I don’t have transportation, I have more privacy at home) are different from those in the main text (17.7% vs 15.9%; 65.0% vs. 58.5).

**Thank you for catching this! Outcomes for this variable should be divided by the proportion of women in the sampling category (prefer to sample at home, n=543). In the abstract and the main text, these proportions were mistakenly reported out of 603. The correct proportions are reported in Table 3. This has now been updated in the manuscript.**

****

**DISCUSSION**

*FGS is a chronic gynaegological condition that afflicts vulnerable women*… What is meant by “vulnerable”?

**In the setting of environmental health emergencies, the WHO describes vulnerability as “the degree to which a population, individual, or organization is unable to anticipate, cope with, resist, or recover from the impact of disasters”. We feel this vulnerability also describes well the plight of women in sub-Saharan Africa in relationship to FGS, as an underreported neglected ailment. Thus, we suggest that the term could be maintained.**

*Current diagnostic strategies are limited as they rely on resources that are seldom available in low-income settings. S*elf-sampling does not contribute significantly to solving this problem, only to minor extent, having the women to perform the sampling themselves instead of a health care provider (e.g a nurse). Other major cost will remain, including lab technicians, equipment, reagents etc.

**Thank you for this input. We agree that many of the other costs will remain and we have attended to this in the manuscript discussion. However a full cost-effective analysis was beyond this pilot work.**

Participants should always be acknowledged.

**Thank you for bringing this oversight to our attention. We have acknowledged the participants.**
